# Prevalence and clinical implications of the rare arc of Bühler using computed tomography angiography and digital subtraction angiography: a systematic review and meta-analysis

**DOI:** 10.3389/fmed.2024.1522292

**Published:** 2025-02-07

**Authors:** Gaowu Yan, Yong Li, Suyu He, Hongwei Li, Morgan A. McClure, Qiang Li, Jifang Yang, Hu Wang, Linwei Zhao, Xiaoping Fan, Jing Yan, Siyi Wu, Wenwen Guo

**Affiliations:** ^1^Department of Radiology, Suining Central Hospital, Suining, China; ^2^Department of Radiology, Shehong Municipal Hospital of Traditional Chinese Medicine, Shehong, China; ^3^Department of Gastroenterology, Suining Central Hospital, Suining, China; ^4^Department of Radiology, The Third Hospital of Mianyang and Sichuan Mental Health Center, Mianyang, China; ^5^Department of Radiology and Imaging, Institute of Rehabilitation and Development of Brain Function, The Second Clinical Medical College of North Sichuan Medical College, Nanchong Central Hospital, Nanchong, China

**Keywords:** arc of Bühler, computed tomography angiography, digital subtraction angiography, meta-analysis, systematic review

## Abstract

**Background:**

Knowledge of the rare arc of Bühler (AOB) is limited but clinically important. At present, there is no publication of systematic review and meta-analysis on AOB in computed tomography angiography (CTA) and digital subtraction angiography (DSA) examinations.

**Objective:**

The objective of this study was to evaluate the pooled prevalence and clinical implications of the AOB by using CTA and DSA examinations.

**Methods:**

The PubMed, Web of Science, Scopus, Embase, Google Scholar, CBM, CNKI, WanFang, VIP, and Baidu Scholar databases were comprehensively searched for AOB-related literature. Stata 17.0 software was used to conduct the meta-analysis.

**Results:**

Eleven publications with 3,837 patients and 65 AOB cases were included. The pooled prevalence of AOB was 1.9% (95% confidence interval: 0.8–3.2%). CTA showed a pooled prevalence of AOB of 2.0% (95% confidence interval: 0.5–4.3%) and DSA showed a pooled prevalence of AOB of 1.8% (95% confidence interval: 0.5–3.9%).

**Conclusion:**

AOB is a rare anatomical variant, with a pooled prevalence of 1.9% in the general population. General surgeons, vascular surgeons, and interventional radiologists should consider its existence when performing relevant abdominal procedures to avoid intraoperative difficulties, visceral organ ischemia or bleeding, and other complications.

## Introduction

The arc of Bühler (AOB) was first described by Bühler in 1904, and it is currently defined as the anastomotic artery between the superior mesenteric artery and the celiac trunk or its branches (1). AOB is a rare anatomical variation that may affect various interventional radiological operations (e.g., interventional embolization of aneurysms) and abdominal surgeries (e.g., pancreaticoduodenectomy) (2, 3). However, most general surgeons, vascular surgeons, and interventional radiologists do not have a sufficient understanding of the anatomy and function of AOB, which may lead to a lack of awareness of potential adverse outcomes (such as iatrogenic injuries) during or after surgeries (4, 5). Therefore, when performing abdominal related surgeries, it is important to keep in mind the existence of AOB.

Computed tomography angiography (CTA) can noninvasively evaluate vascular diseases and anatomical variations in various parts of the body, and digital subtraction angiography (DSA) is currently considered the reference standard to diagnose vascular diseases in all parts of the body (6, 7). As a result, both CTA and DSA are clinically valuable to evaluate the prevalence and clinical implications of AOB.

With the rapid development of evidence-based medicine (EBM), the theory and concept of evidence-based anatomy (EBA) came into being. EBA applies the basic principles and research methods of EBM to the field of anatomy (8, 9). The actual prevalence of AOB remains unclear, with estimates in the literature suggesting it to be less than 3% (1). Thus, this study aimed to use a systematic review and meta-analysis (i.e., EBA) to review and analyse the literature on AOB and evaluate its reported prevalence and clinical implications based on CTA and DSA to guide the diagnosis and treatment of clinically related diseases.

## Materials and methods

### Inclusion and exclusion criteria

Studies of the general population who underwent abdominal CTA or DSA to evaluate the presence of anatomical variations in the abdominal vasculature were included in this systematic review and meta-analysis. The outcome index was the prevalence of AOB. In this present study, AOB was defined as the anastomotic artery between the superior mesenteric artery and the celiac trunk or its branches (1). The exclusion criteria were: (I) reviews, case reports, editorials, comments, and conference abstracts; (II) autopsies of animal or human cadavers; (III) duplicate publications; and (IV) an inability to obtain the full text, incomplete literature data, or data that could not be used to calculate the prevalence of AOB.

According to our previous experience in systematic review and meta-analysis, there are two common types of duplicate publications. The first type is multiple publication of the same research data in different languages. The second type is that some time following the publication of a study, a second study by the same author(s) on the same topic, with a larger sample size, was published. In the second situation, we will exclude the earlier study and only include the latest study with the largest sample size.

### Literature retrieval strategy

The English databases of PubMed, Web of Science, Embase, Scopus, and Google Scholar; and the Chinese databases of CBM (China Biology Medicine Disc), CNKI (China National Knowledge Infrastructure), WanFang, VIP, and Baidu Scholar were all comprehensively searched. All cross-sectional studies on the prevalence of AOB were collected. The time frame for retrieval was from the establishment of each database to September 30, 2024. A combination of subject and free words that were modified based on the characteristics of each database was used for retrieval. Simultaneously, the references included in the study were searched to supplement and obtain relevant data. The searched words included in this study were Bühler, Buhler, arc, Bühler arc, Buhler arc, coeliac, coeliac trunk, celiac, celiac trunk, coeliac artery, celiac artery, celiac axis, celiac axis, trunk, axis, and superior mesenteric artery.

### Literature screening and data extraction

Two researchers (with eight and 10 years of experience in abdominal radiology) independently examined the literature, extracted the data, and cross-checked it. If there were disagreements, they were resolved by discussion or consultation with a third researcher (with 15 years of experience in abdominal radiology). The title of the article was reviewed first throughout the literature screening procedure. Upon eliminating studies that were clearly irrelevant, the abstract and complete text were examined to decide whether or not to incorporate the study into the analysis. If necessary, the authors of the original study were contacted by e-mail or telephone to obtain data that were not reported but were important for this study. First author, publication time (year), country, research type (CTA or DSA), total sample size, number and prevalence of AOB (%), and AOB type were all included in the data.

### Bias risk assessment

The same two researchers who performed the literature screening and data extraction procedures independently evaluated the risk of bias and cross-checked the results. Bias risk assessment was performed by using the “Anatomical Quality Assessment Tool” recommended by the International Evidence-Based Anatomy Working Group (10). The tool consisted of a series of questions in five domains: (I) research objectives and characteristics of research subjects, (II) research design, (III) methodology characterization, (IV) descriptive anatomy, and (V) reporting of results. If all questions in a particular domain were answered ‘yes’, then the risk of bias in that domain was determined to be ‘low’; if any question in a particular domain was answered ‘no’ or ‘unclear’, the risk of bias in this domain was, respectively, determined to be ‘high’ or ‘unclear’.

### Statistical analysis

Data were collected and collated by using an Excel (Microsoft Corp., Redmond, WA, USA) table, and a single-group meta-analysis was performed using the metaprop module of Stata software (version 17.0; StataCorp, College Station, TX, USA). The prevalence of AOB was used as the statistic for effect analysis, and a 95% confidence interval (CI) was provided. Heterogeneity between studies was evaluated using *χ*^2^ tests and I^2^ statistics. *χ^2^* tests (*p* < 0.10) indicated statistical heterogeneity. An I^2^ value of 0–40% was considered “might not be important,” that of 30–60% was considered “may represent moderate heterogeneity,” that of 50–90% was considered “may represent substantial heterogeneity,” and that of 75–100% was considered “considerable heterogeneity” (8). For the meta-analysis, the fixed-effects model was to be applied if there was no statistical heterogeneity among the outcomes. In cases where there was statistical heterogeneity in the data, the source of the heterogeneity was investigated further, and after ruling out the impact of clear clinical heterogeneity, a random-effects model was employed for the meta-analysis. The level of the meta-analysis was set as *α* = 0.05. A subgroup analysis was conducted according to the type of study (CTA or DSA) to further explore the possible factors affecting the AOB prevalence.

## Results

### Literature screening procedures and results

**Figure 1:**
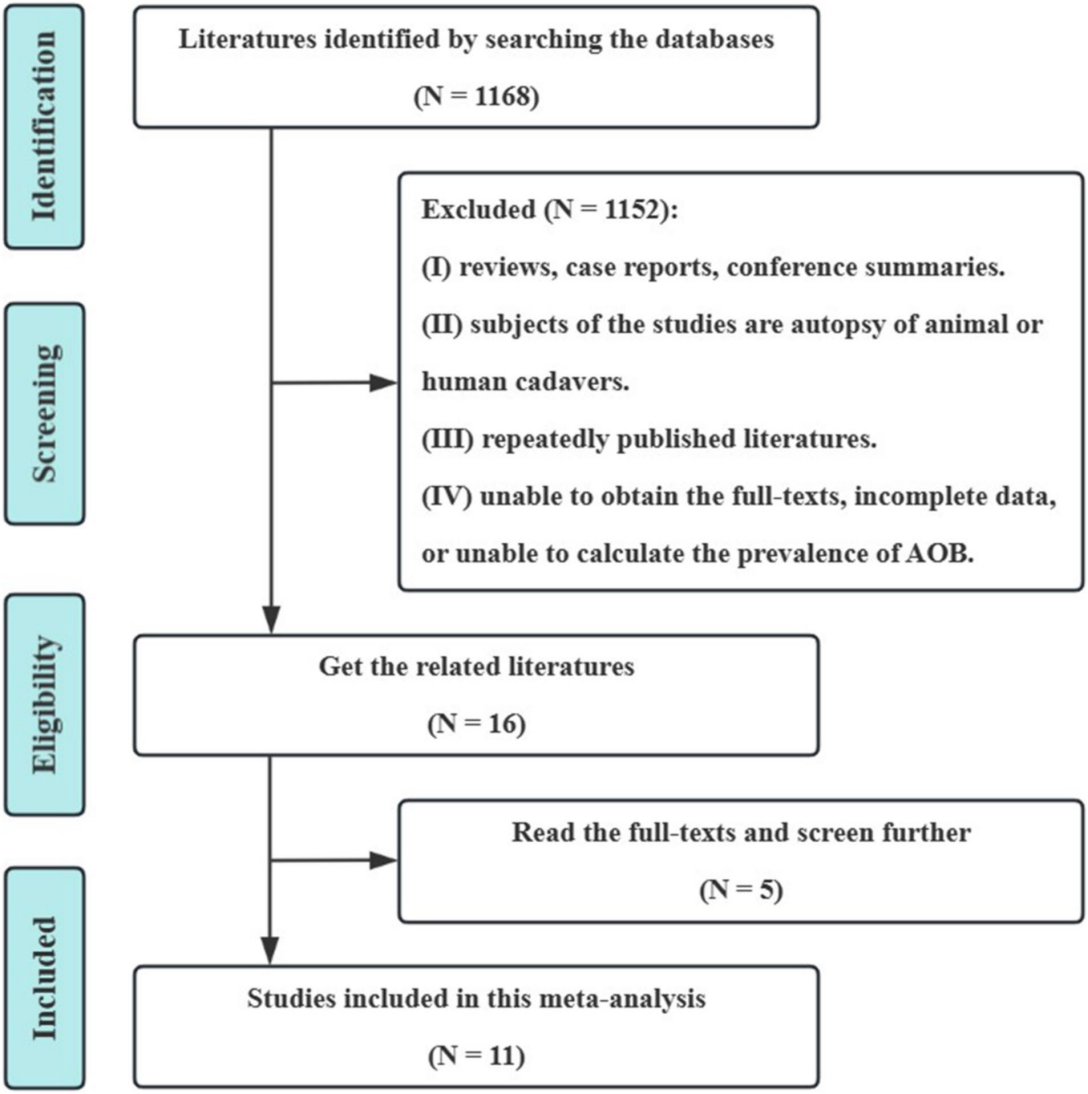
Screening process and literature review results. AOB, the arc of Bühler.

On the basis of the preliminary screening, 1,168 pertinent studies were included. Eleven cross-sectional studies were eventually included following layer-by-layer screening (11–21). These included 3,837 participants (with 65 cases of AOB), of which 1769 were examined using CTA and 2068 were examined using DSA. The literature screening procedure and the results are shown in Figure 1.

### Basic features and bias risk assessment results

**Figure 2:**
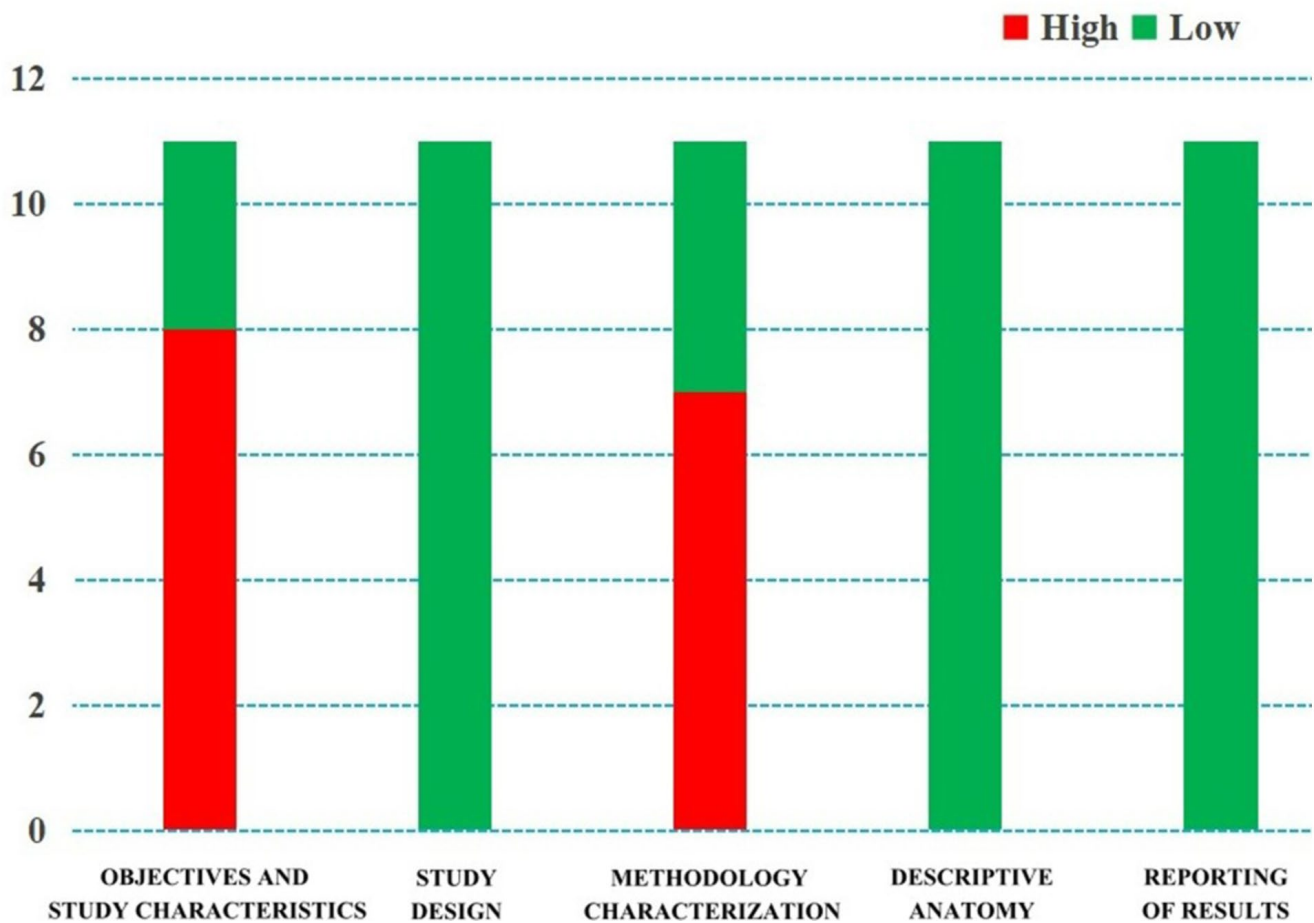
Quality evaluation by the “Anatomical Quality Assessment (AQUA) Tool” for the included studies.

The basic characteristics of the included studies and the results of the bias risk evaluation are presented in Tables 1, 2, respectively. The bias risk of eight studies in terms of “research objectives and characteristics of research subjects” was determined to be ‘high’, as the method of subject selection introduced bias into the study to some extent (*n* = 8), and that of seven studies in “methodology characterization” was determined to be ‘high’ due to the absence of appropriate measures for reducing inter- and intra-observer variability (*n* = 7). The bias risk in the remaining domains was determined to be ‘low’ (Figure 2).

**Table 1:** Basic features and prevalence of AOB in included studies.

Author	Year of publication	Country	Study type	Sample size	AOB (%)	AOB composition
Wicke et al. (11)	1977	Austria	DSA	80	1 (1.25)	Celiac trunk-superior mesenteric artery
Grabbe et al. (12)	1980	Germany	DSA	340	14 (4.12)	Celiac trunk-superior mesenteric artery
Bertelli et al. (13)	1991	Italy	DSA	1,000	3 (0.30)	Celiac trunk-superior mesenteric artery
McNulty et al. (14)	2001	Ireland	DSA	300	3 (1.00)	Celiac trunk-superior mesenteric artery
Saad et al. (15)	2005	America	DSA	120	4 (3.33)	Celiac trunk-superior mesenteric artery
Ferrari et al. (16)	2007	Italy	CTA	60	2 (3.33)	Celiac trunk-superior mesenteric artery, Celiac trunk-splenetic artery
Sureka et al. (17)	2013	India	CTA	600	8 (1.33)	Celiac trunk-superior mesenteric artery
van Petersen et al. (18)	2014	Netherlands	DSA	228	7 (3.07)	Celiac trunk-superior mesenteric artery
Ognjanović et al. (19)	2014	Serbia	CTA	150	4 (2.67)	Celiac trunk-superior mesenteric artery
Farghadani et al. (20)	2016	Iran	CTA	607	2 (0.33)	Celiac trunk-superior mesenteric artery
Lin et al. (21)	2022	China	CTA	352	17 (4.8)	Celiac trunk-superior mesenteric artery and its branches^*^

CTA, computed tomography angiography, DSA, digital subtraction angiography, and AOB, Arc of Bühler. ^*^Celiac trunk-superior mesenteric artery (*n* = 6), splenetic artery-superior mesenteric artery (*n* = 4), common hepatic artery-superior mesenteric artery (*n* = 3), celiac trunk-inferior pancreaticoduodenal artery (*n* = 1), dorsal pancreatic artery-inferior pancreaticoduodenal artery (*n* = 1), celiac trunk-ectopic origin of right hepatic artery (n = 1), and common hepatic artery-middle colonic artery (*n* = 1).

**Table 2:** Basic characteristics of included studies and AQUA quality evaluation.

Author	Objectives and subject characteristics	Study design	Methodology characterization	Descriptive anatomy	Reporting of results
Wicke et al. (11)	High	Low	High	Low	Low
Grabbe et al. (12)	High	Low	High	Low	Low
Bertelli et al. (13)	High	Low	High	Low	Low
McNulty et al. (14)	High	Low	High	Low	Low
Saad et al. (15)	High	Low	High	Low	Low
Ferrari et al. (16)	High	Low	Low	Low	Low
Sureka et al. (17)	Low	Low	Low	Low	Low
van Petersen et al. (18)	Low	Low	High	Low	Low
Ognjanović et al. (19)	High	Low	High	Low	Low
Farghadani et al. (20)	High	Low	Low	Low	Low
Lin et al. (21)	Low	Low	Low	Low	Low

AQUA, anatomical quality assessment tool.

### Results of the meta-analysis

#### Pooled prevalence

**Figure 3:**
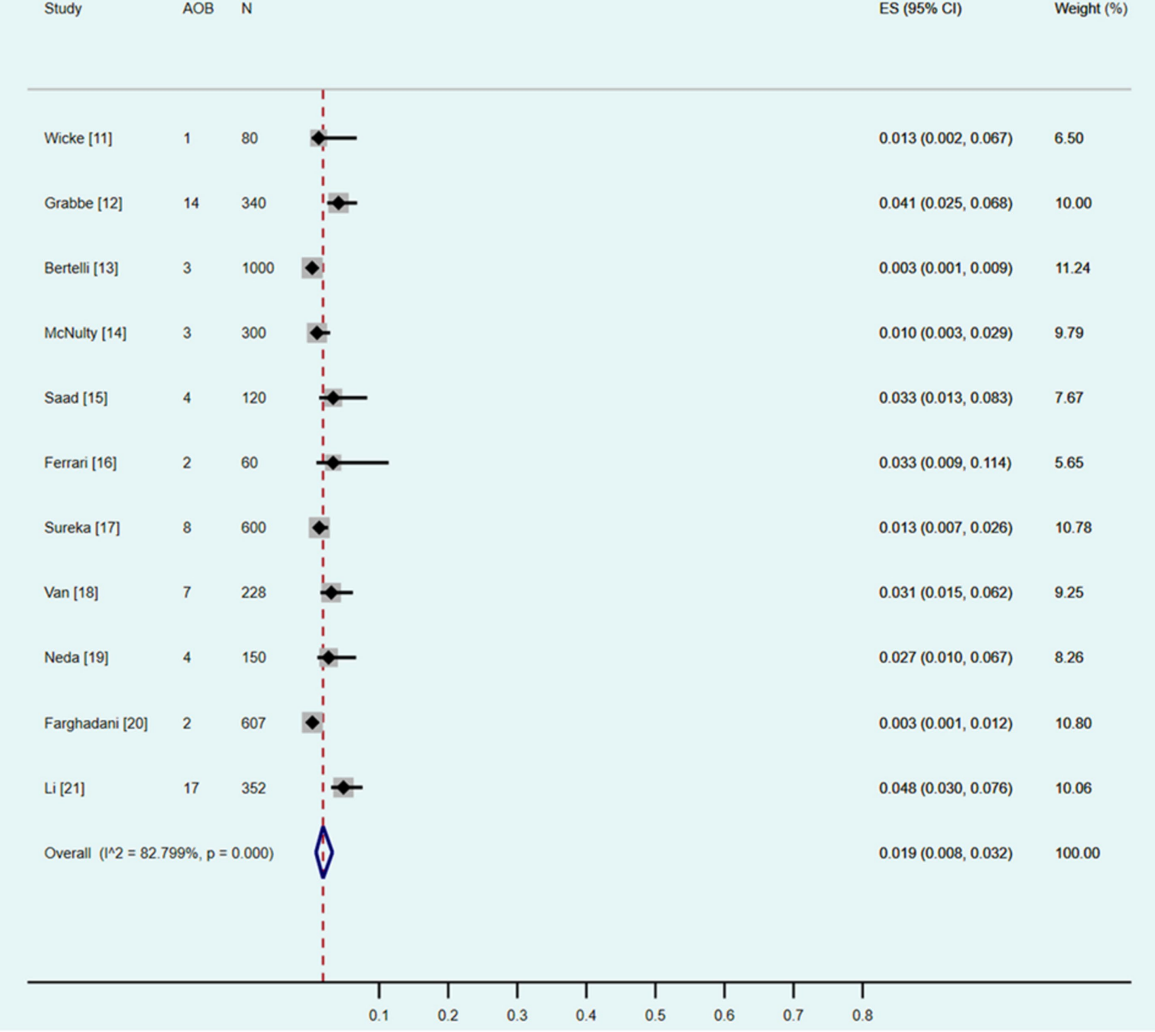
Forest plot showing a pooled incidence of the arc of Bühler (AOB) of 1.9% (95% confidence interval, 0.8–3.2%).

Eleven studies were included (11–21). The *χ*^2^ test identified a *p* value of less than 0.10, and the I^2^ statistic was 82.80%. Both of the results indicated statistically significant heterogeneity between the eleven included studies. The results of the random effect model meta-analysis (Table 3 and Figure 3) showed that the pooled prevalence of AOB was 1.9% (95% CI: 0.8–3.2%).

**Table 3:** Subgroup analysis according to the type of included studies.

Subgroup	Number of studies	Number of patients	Pooled prevalence of AOB: % (95% CI)	I^2^ statistics (%)	*P* value
Overall	11	3,837	1.9 (0.8–3.2)	82.799	< 0.001
CTA	5	1769	2.0 (0.5–4.3)	84.561	< 0.001
DSA	6	2068	1.8 (0.5–3.9)	84.129	< 0.001

CTA, computed tomography angiography, DSA, digital subtraction angiography, and AOB, Arc of Bühler.

### Subgroup analysis

Five CTA and six DSA studies were included into the subgroup analysis (Table 3). The *χ*^2^ tests identified both of the *p* values were less than 0.10, and the I^2^ statistics were 84.56 and 84.13%, respectively. All the results indicated statistically significant heterogeneity between the studies included into the subgroup analysis. According to the subgroup analysis of the included studies with the random effect models, the pooled prevalence of the AOB using CTA was 2.0% (95% CI: 0.5–4.3%), and that using DSA was 1.8% (95% CI: 0.5–3.9%).

### Sensitivity analysis and publication bias test

**Figure 4:**
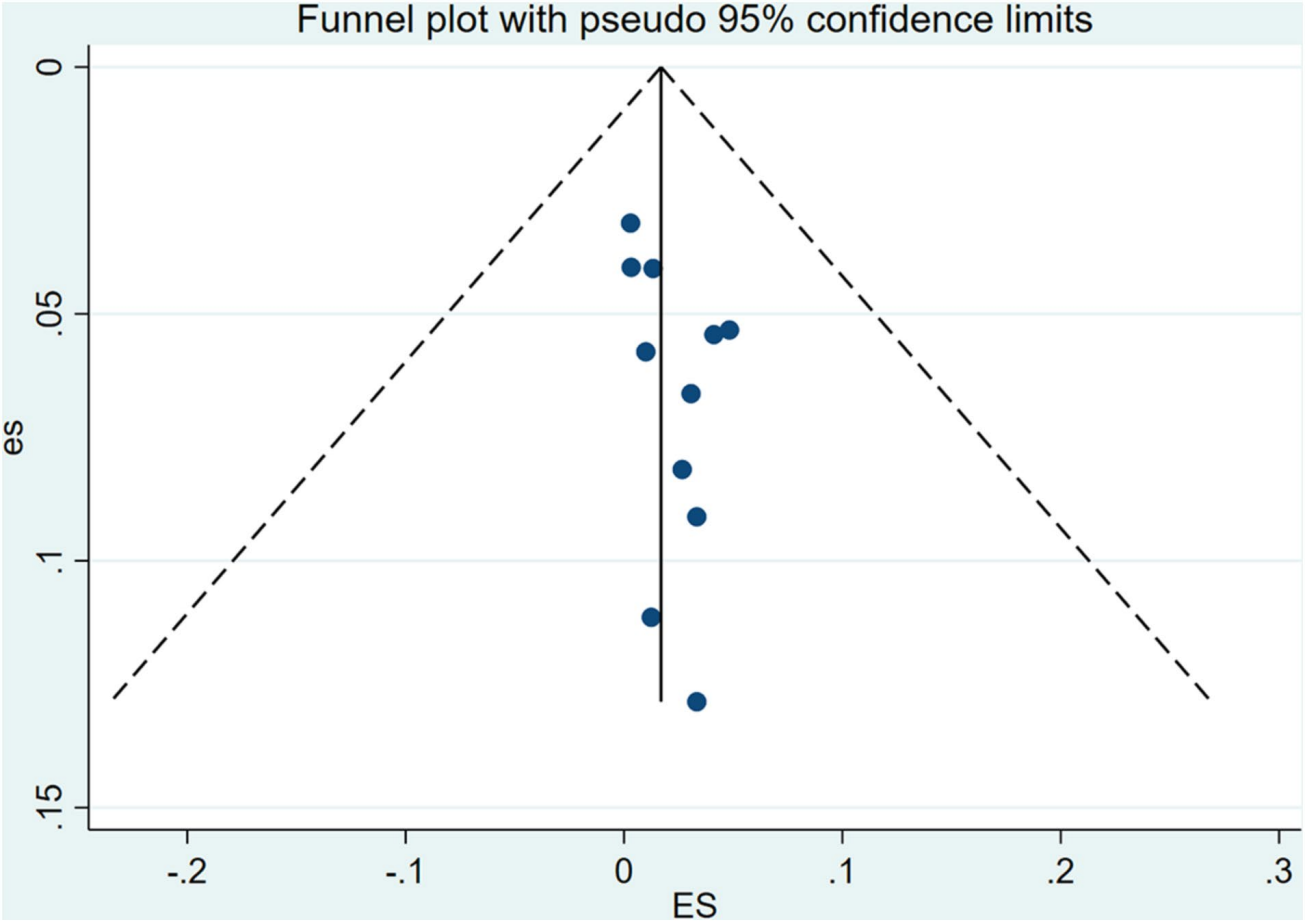
Funnel plot showing no publication bias in this study.

Sensitivity analysis was performed by eliminating individual studies; the results showed no significant change in the combined estimates, suggesting that the results of this study are relatively stable. The publishing bias of the included studies was assessed by using funnel plots, Begg’s test, and Egger’s test (Figure 4). The findings indicated no significant publication bias (*p* > 0.05).

## Discussion

### Definition, prevalence, and anatomical types of the AOB

AOB is a rare anatomical variation. Bühler was the first to describe AOB as “the anastomotic artery between the celiac artery and the middle colonic artery, usually as an additional collateral circulation between the celiac artery and the superior mesenteric artery” (1). With the increase in subsequent reports and a deepening of understanding, current studies have found that such anastomotic vessels can also occur in multiple branches of the celiac trunk and superior mesenteric artery including the following: celiac trunk and superior mesenteric artery, celiac trunk and inferior pancreaticoduodenal artery, celiac trunk and middle colonic artery, celiac trunk and first jejunal artery, celiac trunk and ectopic origin of right hepatic artery, splenetic artery and superior mesenteric artery, common hepatic artery and superior mesenteric artery, common hepatic artery and middle colonic artery, proper hepatic artery and first jejunal artery, gastroduodenal artery and pancreaticoduodenal artery, and pancreatic integument artery and anterior/posterior/inferior pancreaticoduodenal artery (1, 11–21). At present, AOB has been redefined as the anastomotic artery between the superior mesenteric artery and the celiac trunk or its branches (1).

In this meta-analysis, the AOB included in the study was primarily found between the celiac trunk and superior mesenteric artery (11–20), while Li and colleagues (21) reported many other types of AOB (Table 1). The author believes that the reasons may be as follows. First, in the early literature, researchers had insufficient understanding of the AOB, which was only defined as “the anastomotic artery between the celiac artery and the superior mesenteric artery”; thus, the reported frequency of AOB type is fairly low. Second, in subsequent studies, researchers had a deeper understanding of the AOB, which was redefined as “the anastomotic artery between the superior mesenteric artery and the celiac trunk or its branches”; thus the reporting frequency of AOB type was improved.

**Figure 5:**
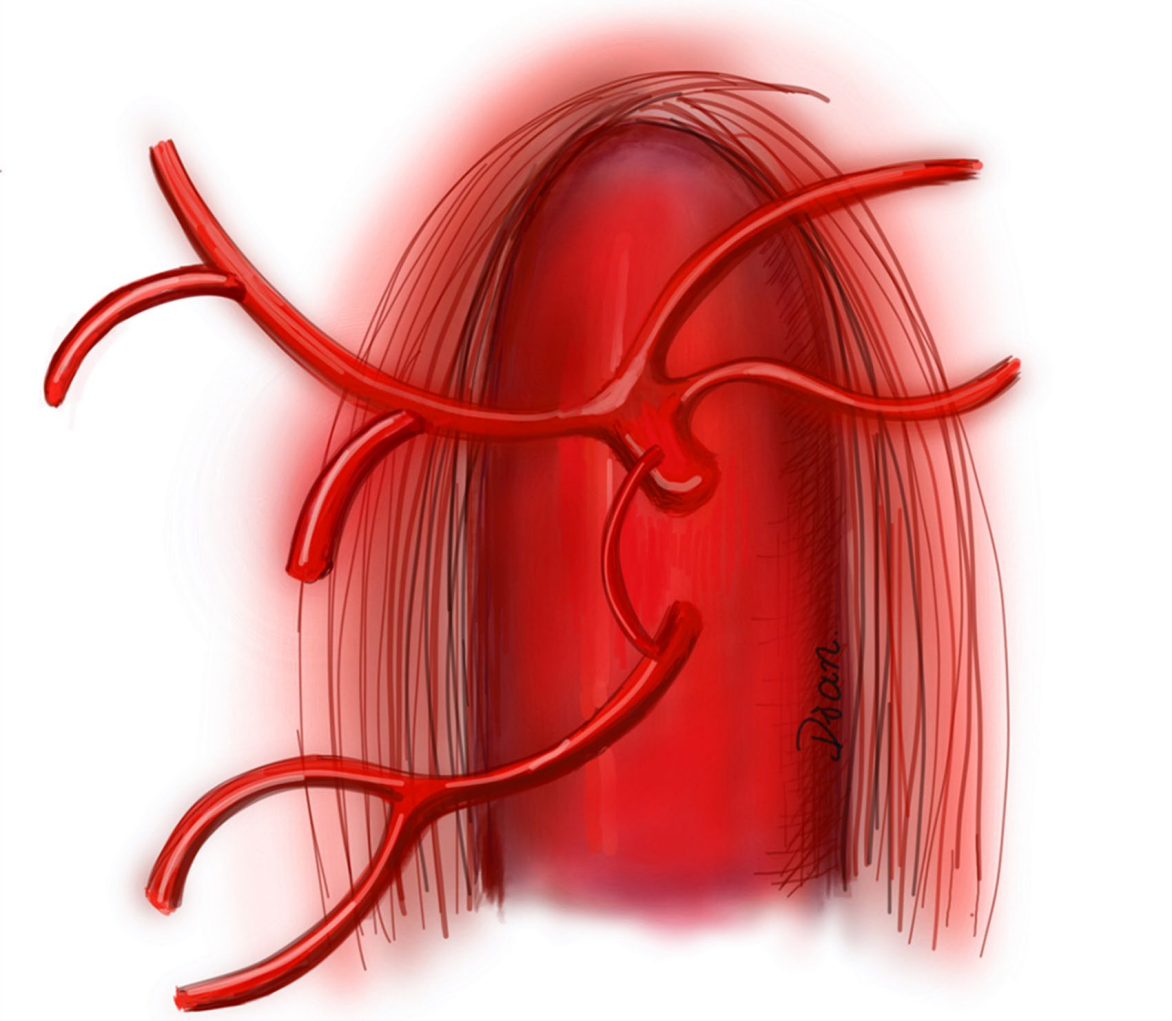
Diagram of the arc of Bühler (24). The arc of Bühler (AOB) is shown as an anastomotic artery between the celiac trunk and the superior mesenteric artery.

In this present meta-analysis, the overall prevalence of AOB was 1.9% (0.8–3.2%). For the subgroup analysis according to the type of inclusion study (Table 3), the pooled prevalence of AOB detected by CTA was 2.0% (95% CI: 0.5–4.3%) and that detected by DSA was 1.8% (95% CI: 0.5–3.9%). This is consistent with previous reports by Dubel et al. (22) and Xiao et al. (23) who demonstrated that the prevalence of AOB in the general population is less than 4.0% (Figure 5) (24).

### Embryological mechanism of AOB

**Figure 6:**
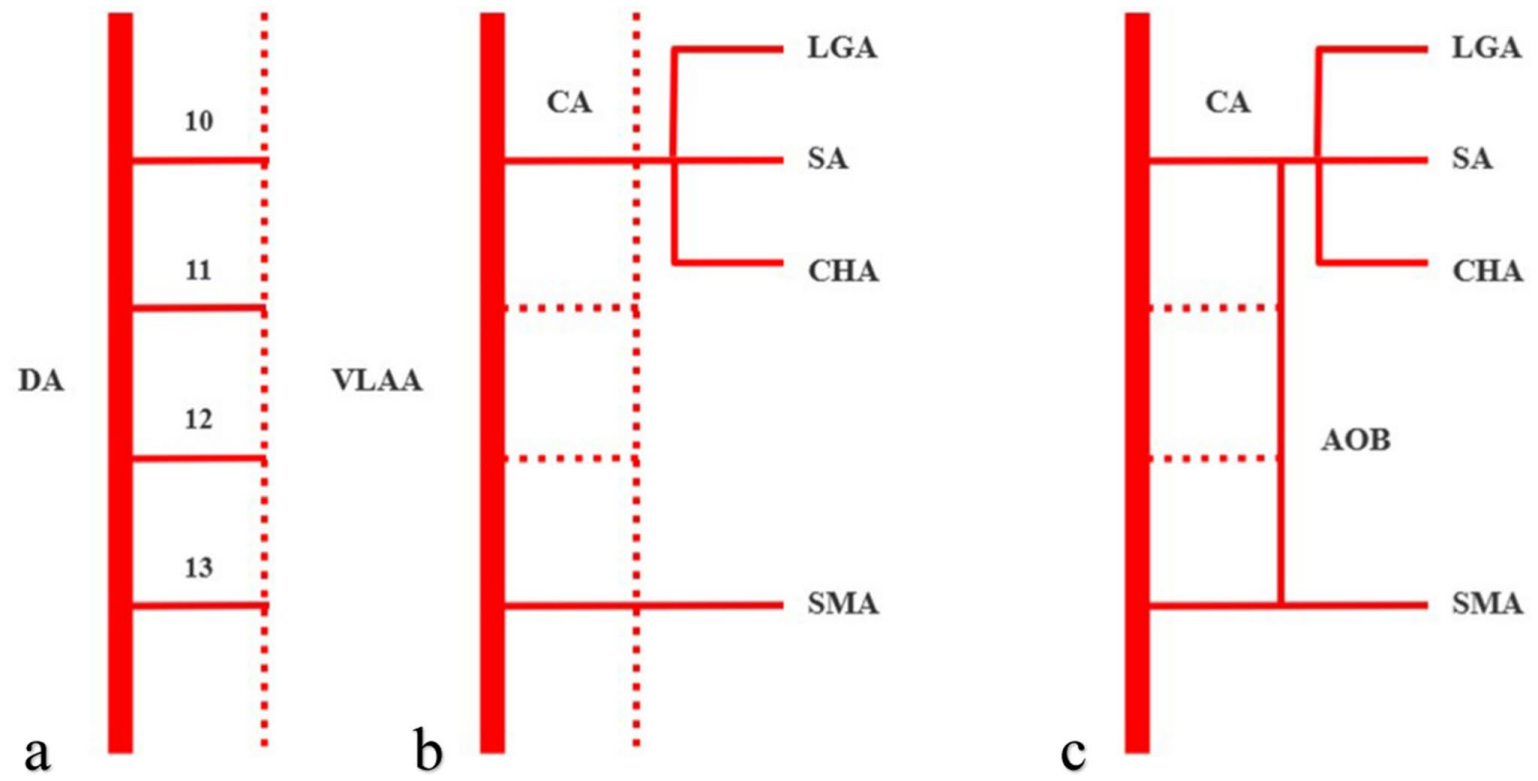
Arc of Bühler (AOB) schematic diagram of the embryological mechanism, with solid lines representing persistent blood vessels and dotted lines representing degenerated blood vessels. The 10th–13th ventral segmental arteries communicate temporarily through ventral longitudinal anastomoses **(a)**. When the 10th and 13th ventral segmental arteries remain, then the celiac trunk and superior mesenteric artery will originate separately from the abdominal aorta **(b)**. An AOB may form when the ventral longitudinal anastomotic artery persists **(c)**. DA, dorsal aorta, VLAA, ventral longitudinal anastomoses artery, CA, celiac axis, LGA, left gastric artery, SA, splenetic artery, CHA, common hepatic artery, and SMA, superior mesenteric artery.

The MacKay arch theory and Handler’s longitudinal anastomosis are two commonly used models to explain anatomical variation in abdominal aortic branches (25). According to the aforementioned model (25), the 10th–13th ventral segmental arteries (which develop into the left gastric artery, splenetic artery, common hepatic artery, and superior mesenteric artery, respectively) originate from the dorsal aorta in the initial stages of embryonic development. They communicate temporarily through ventral longitudinal anastomoses (Figure 6a). Under normal circumstances, the 11th and 12th ventral segmental arteries and ventral longitudinal anastomotic arteries degenerate and disappear, while the 10th and 13th ventral segmental arteries remain, finally forming the celiac trunk and the superior mesenteric arteries. Thus, both the celiac trunk and superior mesenteric artery originate separately from the abdominal aorta (Figure 6b). If the 13th ventral segmental artery and ventral longitudinal anastomotic artery persist and the 10th–12th ventral segmental arteries degenerate, the celiac trunk and superior mesenteric artery are formed. An AOB may form when the ventral longitudinal anastomotic artery persists (Figure 6c). These embryological mechanisms were further clarified in later studies (26–28).

### Physiological function and clinical symptoms of AOB

The main physiological function of AOB is to act as a collateral circulation between the celiac trunk and the superior mesenteric artery. Saad et al. (15) used a 1.67 mm ductus arteriosus as a reference and estimated the AOB diameter to be 1.5–2.5 mm. In another study, Grabbe and Bühler (12) estimated that the AOB diameter was 2.0–7.0 mm. Therefore, the AOB is usually very small, and the blood flow may be negligible under normal conditions; however, the hemodynamic behavior of the AOB may change in cases of stenosis or occlusion of the celiac trunk or superior mesenteric artery. In such cases, blood circulates through the collateral circulation of the AOB, ensuring blood circulation to the abdominal organs. However, this collateral circulation mechanism may also lead to the formation of AOB aneurysms because increased local blood flow may lead to increased arterial pressure, thickening of local arterial walls, and the formation of true aneurysms (29). AOB can also form an arterial shunt between the celiac trunk and superior mesenteric artery, resulting in decreased blood flow through the common hepatic artery (30).

The vast majority of patients with AOB did not have any clinical symptoms (86.79%), with it being incidentally detected on CTA or DSA. A few patients (13.21%) may have had epigastric discomfort, postprandial pain, gastrointestinal bleeding, obstructive jaundice, bleeding after endoscopic retrograde cholangiopancreatography, intraabdominal or retroperitoneal bleeding due to pressure phenomena, endovascular hemorrhage, or ruptured aneurysms (1, 23, 31–34).

### Clinical correlation of AOB

The anatomy of the retropancreatic space is important during pancreaticoduodenectomy. The tissue structures in this area include the celiac trunk, superior mesenteric artery, portal vein, lymph nodes, and nerves. During pancreaticoduodenectomy, accidental injury to the AOB may lead to iatrogenic damage, such as abdominal organ ischemia or bleeding (35, 36). For interventional radiologists, in some cases (e.g., abdominal trunk occlusion), an unobstructed AOB can also become an alternative route for some abdominal surgeries, or even “an anatomical variation that saves patients’ lives” (37, 38). Shah et al. (37) reported a patient who underwent AOB intubation chemoembolization for hepatocellular carcinoma due to abdominal trunk occlusion.

In another study, Nikolaos et al. (38) reported a patient with complex type B aortic dissection, whose abdominal trunk was almost completely occluded due to the involvement of the dissection, and faced a huge risk of abdominal organ malperfusion syndrome. However, after careful examination by the physicians, it was discovered that there was an AOB anatomical variation between the common hepatic artery and the superior mesenteric artery in the patient, which allowed for the maintenance of blood supply to the abdominal organs. As a result, the patient ultimately underwent endovascular repair surgery for aortic dissection to save his life.

Using PubMed for a literature retrieval with the keywords “arc of Bühler,” “arc of Buhler,” “Bühler,” and “Buhler” (December 20, 2024), we identified a few publications on AOB in some clinical scenarios. For example, Quaretti et al. (39) reported a case of large Buhler aneurysm in the context of chronic celiac trunk occlusion, which was successfully treated by means of covered stent assisted coil embolization; Abouzaid et al. (40) presented a unique AOB variant that connecting the arteries of the foregut, midgut, and hindgut, which was more complex than what had previously been reported; and in another study, Ehemann and Kim (41) reviewed an extremely rare vascular complication of extracorporeal shock wave lithotripsy (ESWL) pseudoaneurysm of AOB, with only one other case recorded in the literature. Publications regarding AOB indexed in the PubMed from 2020 to 2024 have been summarized in Table 4 (*n* = 10).

**Table 4:** Publications regarding arc of Bühler or Buhler indexed in the PubMed from 2020 to 2024 (*n* = 10).

Author/References	Year	Gender	Age (years)	Clinical presentations	AOB composition	Main highlights	Result
Huang et al. (2)	2024	Female	62	Two months of recurrent abdominal distension and postprandial pain	Celiac trunk-superior mesenteric artery	Revascularization of a superior mesenteric artery ostial occlusion via the arc of Buhler	Resolution
Quaretti et al. (39)	2024	Male	47	During rehabilitation following motorcycle trauma and vertebral surgery	Celiac trunk-superior mesenteric artery	Covered stent assisted coil embolization of large Buhler aneurysm in setting of chronic celiac trunk occlusion	Resolution
Abouzaid et al. (40)	2023	Male	76	Cadaveric donor	Celiac trunk-superior mesenteric artery	A unique arc of Bühler variant connecting the arteries of the foregut, midgut, and hindgut	NA
Ehemann and Kim (41)	2023	Male	70	Right mid-pole renal calculus	Celiac trunk-superior mesenteric artery	Rare vascular complication of ESWL pseudoaneurysm of arc of Buhler	Resolution
Padar et al. (31)	2023	Female	77	Sudden onset of pleuritic chest pain	Gastroduodenal artery and the first branch of the superior mesenteric artery	Unexpected finding of arc of Buhler with celiac artery stenosis during workup for a suspected pancreatic lesion	Resolution
Rathod et al. (29)	2022	Male	79	Cadaveric donor	Celiac trunk-superior mesenteric artery	A case of abnormally dilated and tortuous arc of Buhler and pancreaticoduodenal arteries in the absence of celiac trunk stenosis	NA
Schumacher et al. (35)	2022	NA	66	Painless jaundice and pathologic elevated cholestasis parameters	Common hepatic artery-superior mesenteric artery	A significant vascular variant in oncologic pancreaticoduodenectomy	Resolution
Manta et al. (44)	2022	Male	60	NA	Celiac trunk-the third jejunal artery	Hexafurcated coeliac trunks, trifurcated common hepatic artery, and a new variant of the arc of Bühler	NA
Incarbone et al. (45)	2021	Female	71	An ischemic stroke caused brain death	Celiac trunk-superior mesenteric artery	Discovery of a rare variant of the arc of Bühler during liver procurement	NA
Nikolaos et al. (38)	2020	Male	54	Sudden, severe, and generalized abdominal pain	Common hepatic artery-superior mesenteric artery	Arc of Buhler can be a lifesaving anatomic variation	Resolution

AOB, arc of Bühler or Buhler; ESWL, extracorporeal shock wave lithotripsy; NA, not available.

### Limitations and future directions

According to the guideline on conducting a systematic review and meta-analysis of anatomical study published by Henry et al., the heterogeneity in anatomical meta-analysis is almost always high (8). As such, due to the intrinsic heterogeneity in anatomical studies and for the purpose of maximizing the validity of the results, we used a random-effects model in our study. In our study, the *χ*^2^ test identified a *p* value of less than 0.10, and the I^2^ statistic was 82.80% for the eleven included studies. Both of the results indicated statistically significant heterogeneity between the studies.

The sources of heterogeneity in anatomical meta-analysis studies should always be explored. To probe them, according to the guideline published by Henry et al., subgroup analysis and sensitivity analysis can be performed (8). In our study, the subgroup analysis was performed by the modalities of the included studies (i.e., CTA *vs.* DSA). Other subgroup analyses, such as by geographical distribution of the studies (seven studies were performed in Europe, while the other four studies were performed in America, India, Iran, and China, respectively), or by gender, age, laterality, and side (left *vs*. right) were not conducted because the data were not applicable. Using confidence intervals (CIs) to assess for statistically significant differences between the CTA and DSA subgroups, an overlap between the two CIs (Table 3) was present. We may consider that the differences between the two groups were statistically insignificant. We also performed a sensitivity analysis to help explore the sources of heterogeneity in our study. The findings of the sensitivity analysis were robust to decisions made in the process of performing the meta-analysis. As a result, we may attribute the heterogeneity of our study to the inherent factors related to the complexity of the human body and diversity.

For methodology characterization and reporting among the included studies, since the concept of evidence-based anatomy was introduced by Henry et al. (8) and Yammine (9) it has emphasized the need for original anatomical studies with high clarity, transparency, and comprehensiveness in reporting. In our study, we used the “Anatomical Quality Assessment (AQUA) Tool” to evaluate the quality of the eleven included studies. The results showed that eight studies had high risk of bias in “objective (s) and subject characteristics” and seven studies had high risk of bias in “methodology characterization.” Tomaszewski et al. (42) and Ottone et al. (43) introduced a checklist of reporting items that should be addressed by authors of original anatomical studies (i.e., the “Anatomical Quality Assurance (AQUA) Checklist”). In this checklist, twenty-nine items, crucial for reporting anatomical studies, have been designed and arranged. The items consist of eight sections, which include title (one item), abstract (one item), introduction (two items), methodology (twelve items), results (five items), discussion (four items), conclusions (one item), and other information (three items). For studies reporting anatomical findings in the future, we would highly recommend using this checklist as a reference.

For future directions, the number and quality of the currently included eleven studies are limited. This indicates the necessity for more investigations involving a large number of subjects, so as to provide a more precise evaluation of the prevalence of AOB, and improve consciousness among general surgeons, vascular surgeons, and interventional radiologists. When preparing to report anatomical findings in the future, we would also highly recommend the researchers involved to use the AQUA checklist as a reference.

## Conclusion

AOB is a rare anatomical variation, with a pooled prevalence of approximately 1.9% in the general population. Although rare, such cases may play a significant role in general surgery, vascular surgery, and interventional radiology. General surgeons, vascular surgeons, and interventional radiologists should consider the existence of AOB when performing associated abdominal operations to avoid complications such as difficulty in the operation, abdominal organ ischemia, or bleeding. Higher-quality, larger-sample studies are required to corroborate the above conclusions because of the restricted number and quality of the included studies in this meta-analysis.

## Data Availability

The datasets presented in this article are not readily available because the raw data supporting the conclusions of this article will be made available by the authors, without undue reservation. Requests to access the datasets should be directed to yangaowu1989@163.com.
